# Novel signaling collaboration between TGF-β and adaptor protein Crk facilitates EMT in human lung cancer

**DOI:** 10.18632/oncotarget.8314

**Published:** 2016-03-24

**Authors:** Aiman Z. Elmansuri, Mishie A. Tanino, Roshan Mahabir, Lei Wang, Taichi Kimura, Hiroshi Nishihara, Ichiro Kinoshita, Hirotoshi Dosaka-Akita, Masumi Tsuda, Shinya Tanaka

**Affiliations:** ^1^ Department of Cancer Pathology, Hokkaido University Graduate School of Medicine, Sapporo, Japan; ^2^ Department of Translational Pathology, Hokkaido University Graduate School of Medicine, Sapporo, Japan; ^3^ Department of Medical Oncology, Hokkaido University Graduate School of Medicine, Sapporo, Japan

**Keywords:** Crk, EMT, lung cancer, small G protein, TGF-β

## Abstract

The signaling adaptor protein Crk has been shown to play an important role in various human cancers. However, its regulatory machinery is not clear. Here, we demonstrated that Crk induced EMT in A549 human lung adenocarcinoma cells through differential regulation of Rac1/Snail and RhoA/Slug, leading to decreased expression of E-cadherin and increased N-cadherin, fibronectin, and MMP2 expression. Cancer cells with mesenchymal features produced TGF-β and also increased the levels of TGF-β receptor. TGF-β increased the endogenous levels of Crk and also augmented Crk-dependent expression of Snail and Slug, and conversely TGF-β receptor inhibitor suppressed the levels of Snail and Slug. Overexpression of Crk was observed at the invasive front of human lung cancer tissues and was significantly associated with poor prognosis. Thus, TGF-β and Crk collaborate to form a positive feedback loop to facilitate EMT, which may lead to the malignancy of human cancers possibly being affected by their microenvironment.

## INTRODUCTION

Crk is a signaling adaptor protein comprising SH2 and SH3 domains, and was originally identified as an avian sarcoma virus encoding oncogene product [1]. Human Crk comprises Crk-I (SH2-SH3) and Crk-II (SH2-SH3n-SH3c), which are splice variants of a single gene [2]. Crk transmits signals from growth factor receptors or cell adhesion molecules by SH2 domain-dependent binding to tyrosine-phosphorylated kinases or components of focal adhesions such as p130^Cas^ or paxillin. Downstream small GTPases including Ras, Rac, and Rap are activated through downstream targets of the SH3 domain of Crk, such as guanine nucleotide exchange factors (GEFs) including C3G, Dock180, and Sos, to regulate cell motility, proliferation, and invasion [3]. Overexpression of Crk is observed in various human cancers, but the precise regulatory mechanism is still unknown [4].

In the process of human cancer progression, the epithelial mesenchymal transition (EMT) is known to be the initial step of invasion and metastasis, and is eventually related to cancer stemness [5–8]. Several transcription factors such as Snail, Slug, Twist, and ZEB1 are involved in EMT features defining the decreased E-cadherin expression and elevated levels of N-cadherin, fibronectin, and MMP2, resulting in specific mesenchymal morphology and function [5, 9–11]. Tumor microenvironment comprising surrounding fibroblasts, endothelial cells, and tumor-associated macrophages, contributes to the EMT through the production of TGF-β and FGF, together with exhibition of tumor heterogeneity and formation of cancer stem cell niches associated with therapeutic resistance against irradiation, chemotherapy, and molecular-targeted reagents [12–14].

In this study, to uncover the mechanism of human cancer cell acquisition of mesenchymal features that cause therapy resistance, we focused on the role of Crk signaling adaptor protein and found that it induced EMT, resulting in the production of TGF-β, which increased Crk levels to form a positive feedback loop. Thus, these two factors cooperatively induced the EMT phenotype in A549 human lung cancer cells, and hence the presence of this TGF-β/Crk signaling axis-induced EMT relates to poor prognosis of lung cancer patients.

## RESULTS

### Crk induced EMT in A549 human lung carcinoma cells

**Figure 1:**
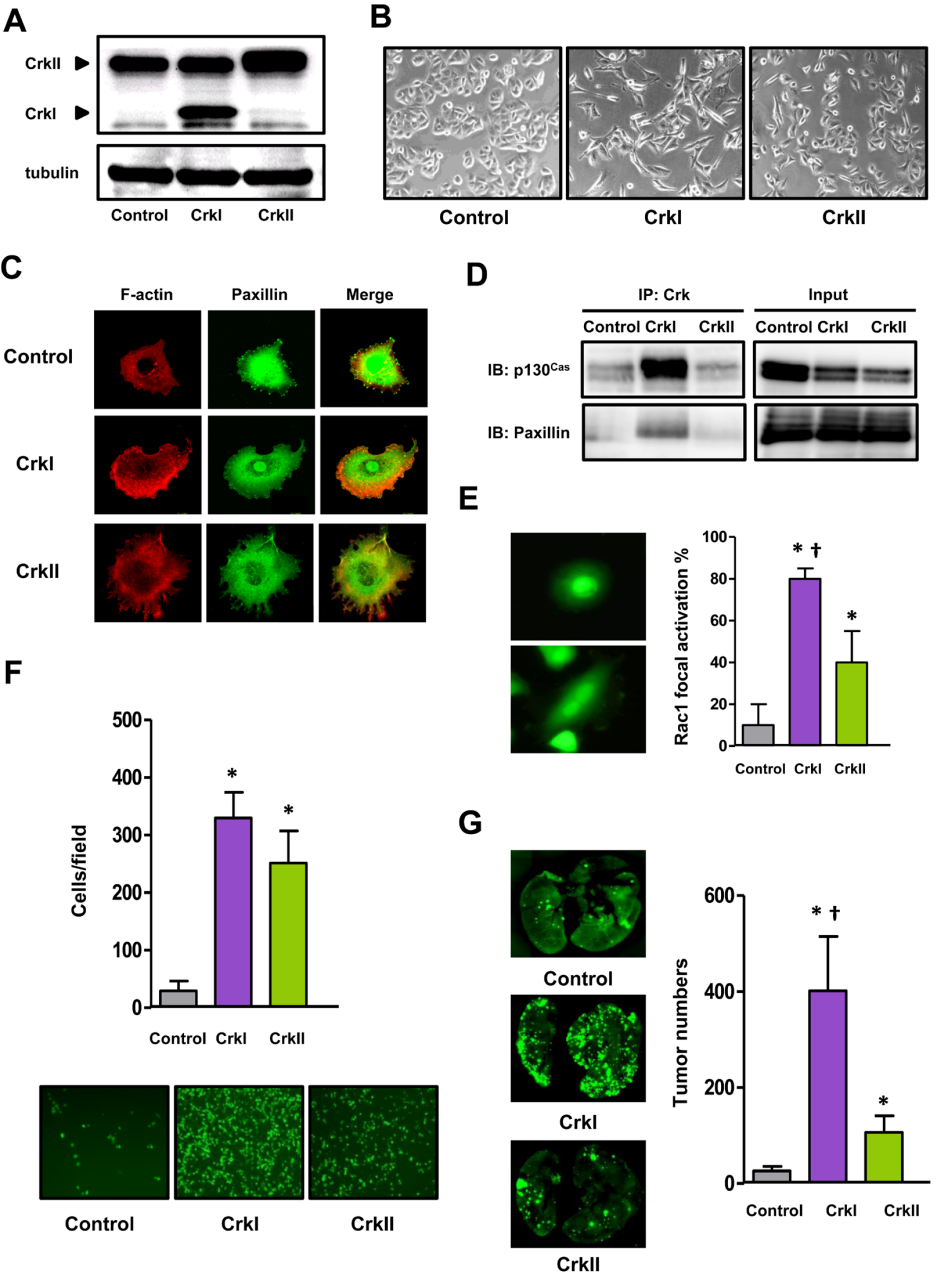
Crk induced EMT in human lung cancer A549 cells **A.** Immunoblotting of Crk levels in A549 cells. The molecular weights of CrkI and CrkII at 28 and 40 kDa, respectively, are indicated by arrow heads. Tubulin was used as an internal control. **B.** Phase-contrast microscopic analysis of Crk-expressing A549 cells. **C.** Immunofluorescence of actin (red) and paxillin (green) of Crk-expressing A549 cells. **D.** Association between Crk and p130^Cas^ or paxillin by immunoprecipitation and immunoblotting. Input indicates total cell lysates. **E.** Immunofluorescence of activated Rac1 in Crk-expressing A549 cells. GST-PAK-RBD was used as a probe, and the proportion of positive cells with membrane ruffling was counted and displayed with control cells (gray bar), and CrkI- (purple bar) and CrkII- (green bar) expressing cells. *P < 0.05 *versus* control cells. ^†^*P* < 0.05 *versus* CrkII cells. Values are means±SD from three independent experiments. **F.** Trans-endothelial assay for evaluation of motility of A549 cells expressing CrkI (purple bar) and CrkII (green bar). Bottom panels are the representative photographs. *P < 0.05 *versus* control cells. Values are means±SD from three independent experiments. **G.** Tail-vein injection assay for evaluation of the distant metastasis ability of A549 cells expressing CrkI (purple bar) and CrkII (green bar). Whole-mount GFP fluorescent images of lung metastasis from NOD mice at 28 days after tail vein injection with indicated A549 cells. The numbers of metastatic nodules were counted (right). *P < 0.05 *versus* control cells. ^†^*P* < 0.05 *versus* CrkII cells. Values are means±SD from eight mice/group for each type of cells.

Correlation of Crk expression and aggressive features has been reported in various human cancers [15, 16], particularly lung cancers [16, 17]. Therefore, to investigate the precise role of Crk in human malignancy, we expressed either CrkI or CrkII in A549 human lung carcinoma cells (Figure 1A). To avoid bias introduced by the differential endogenous levels of Crk-related proteins, the A549 cell line was confirmed to include standard levels of endogenous Crk targets compared with other cell lines (Supplementary information, Figure S1). CrkI- and CrkII-expressing cells exhibited a mesenchymal morphology (Figure 1B) including lamellipodia and filopodia, respectively (Figure 1C), and in particular CrkI-expressing cells showed formation of a complex between CrkI and phosphorylated p130^Cas^ and paxillin (Figure 1D).

Correlating with this mesenchymal morphology, an increase in the active form of RhoA was observed in CrkI-overexpressing cells by pull-down assay (Supplementary information, Figure S2A). As Crk failed to elicit Rac1 activation probably because of masking of the subcellular activation of Rac1 (Supplementary information, Figure S2B) as evaluated by pull-down assay using whole cell lysates of cancer cells, we thus carefully examined the localization of active Rac1 by immunofluorescence using its target PAK1, and found significant increases in ruffling-specific activation of Rac1 observed in Crk-expressing cell lines (Figure 1E and Supplementary information, Figure S2C). In fact, both CrkI- and CrkII-expressing cells possessed enhanced cellular motility and invasiveness, as measured by trans-endothelial (Figure 1F), wound healing, transwell, and Matrigel-invasion assays (Supplementary information, Figure S3A-S3C). Mice tail vein injections of CrkI- and CrkII-expressing cells clearly formed enhanced numbers of metastatic nodules in the lung (Figure 1G). In these invasion-associated assays, CrkI may possess higher activity than CrkII, and correlating with these results, several tyrosine kinases and related proteins such as Met, Gab1, and Src were clearly phosphorylated in CrkI-expressing cells (Supplementary information, Figure S4). Crk expression did not alter cell proliferation potential, as measured by cell counting, BrdU and MTT assay (Supplementary information, Figure S5A-S5C). These data suggest that both CrkI and CrkII induced EMT in human lung cancer cells.

### Crk induced EMT through small GTPases Rac1 and RhoA

**Figure 2:**
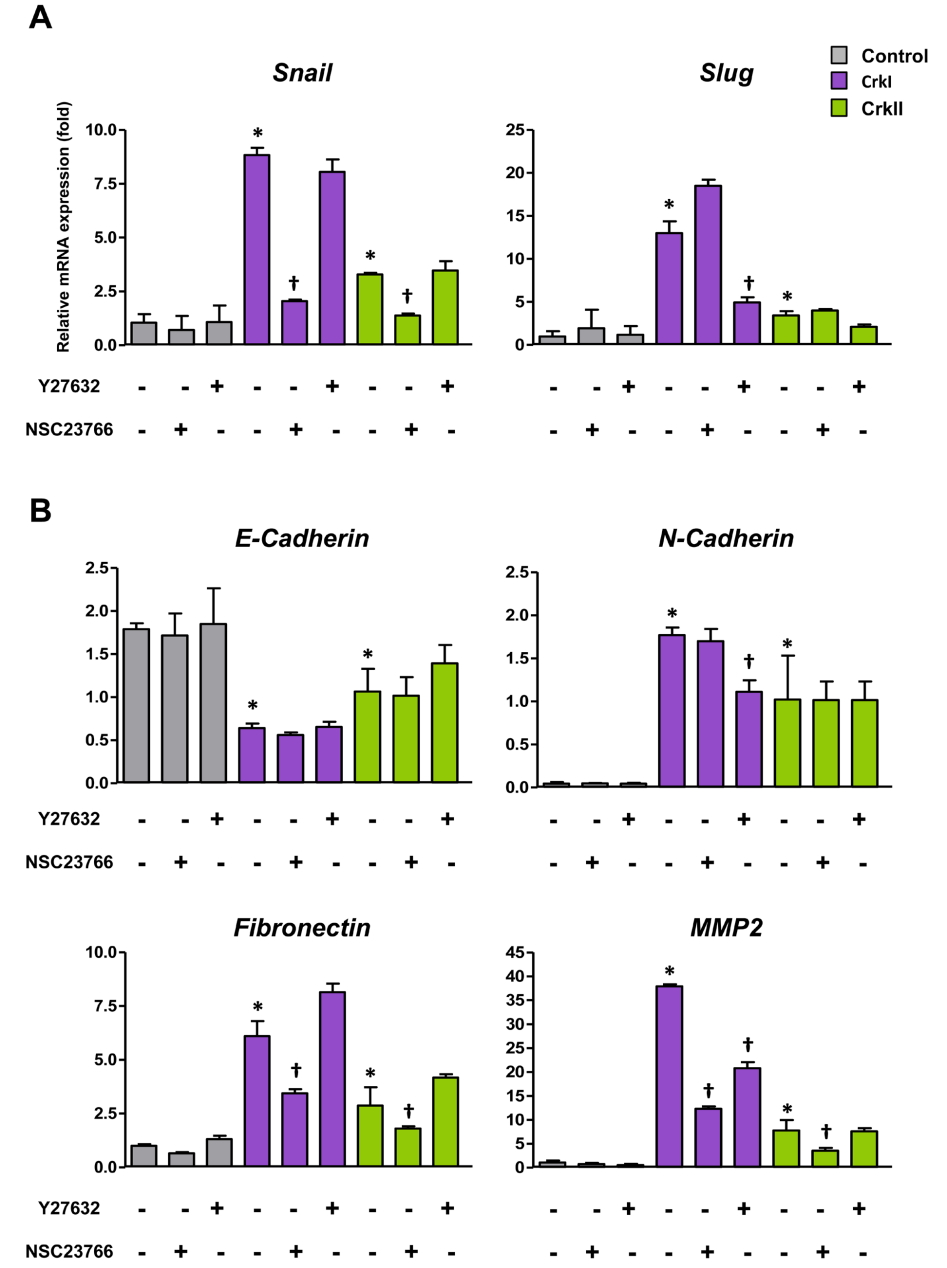
CrkI and CrkII overexpression induces EMT *via* distinct regulation of Rac1 and RhoA **A.** qRT-PCR analysis of mRNA expression of Snail and Slug. Effects of ROCK inhibitor Y27632 (10 μM) and Rac1 inhibitor NSC23766 (50 μM) were also analyzed. **B.** qRT-PCR analysis of mRNA expression of E-cadherin, N-cadherin, fibronectin, and MMP2. Values are means±SD from three independent experiments. *P < 0.05 *versus* control cells. ^†^*P* < 0.05 *versus* untreated cells.

Corresponding to the Crk-induced EMT, both CrkI and CrkII induced expression of EMT-associated representative transcription factors such as *Snail* and *Slug* together with the EMT markers *fibronectin* and *MMP2* (Figure 2A and 2B). A cadherin switch, which exhibits decreased *E-cadherin* and increased *N-cadherin*, was also observed (Figure 2B).

Because Crk bound to GEFs functions as an upstream activator for small GTPases [3], the involvement of Rac1 and RhoA in Crk-mediated EMT was further analyzed, and inhibitors of Rac1 and ROCK (Rho-associated kinase), NSC23766 and Y27632, respectively, were found to specifically suppress the expression levels of *Snail* and *Slug*, respectively (Figure 2A). ROCK inhibitor mainly downregulated *N-cadherin*, whereas Rac1 inhibitor predominantly suppressed the levels of *fibronectin* and *MMP2* (Figure 2B). Gelatin zymography also confirmed Rac1 inhibitor-mediated decreases in MMP2 activity (Supplementary information, Figure S6). These data suggest that Crk-induced EMT was mediated by Rac1 and RhoA, with specific combinations of Rac1/Snail and RhoA/Slug contributing to differential expression of EMT-associated molecules.

### Expression of Crk is induced by various growth factors including TGF-β

**Figure 3:**
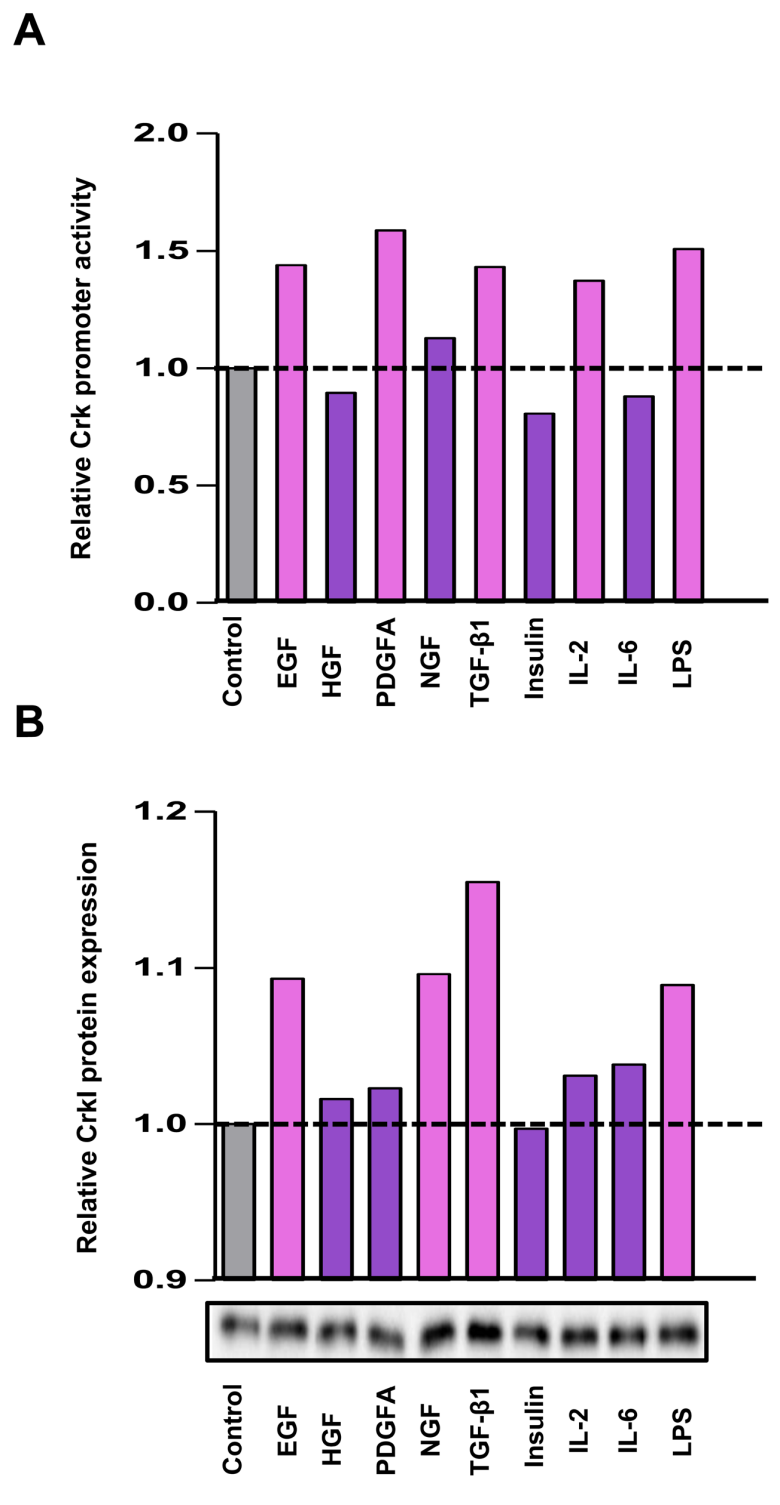
Various stimuli enhanced Crk expression **A.** Luciferase assay for the *Crk* promoter. Serum-starved A549 cells were stimulated with various growth factors and cytokines indicated below the panel, and dual-luciferase assays were performed at 24 h after stimulation. The relative promoter activities of *Crk* were displayed as bar graph. **B.** Expression levels of CrkI were analyzed by immunoblotting. After 48 h, expression levels of CrkI protein were examined. The results of immunoblotting are displayed as bar graph. Gray bar indicates control arbitrarily designated as 1.0, and a significant increase is indicated by pink color and a non-significant increase as purple color.

It has been reported that various human cancers have increased levels of Crk that play essential roles in malignant progression [18–20], but the regulatory mechanism of Crk expression is unclear. As the various growth factors secreted from cancer cells and surrounding stromal cells may regulate cancer cell proliferation in the tumor microenvironment, we examined whether growth factors including EGF, HGF, PDGFα, NGF, TGF-β, and insulin, and several cytokines such as IL-2, IL-6, and LPS, can induce Crk expression. Among them, EGF, TGF-β, and LPS were found to enhance both the promoter activity of Crk as measured by luciferase assay (Figure 3A), and CrkI protein levels (Figure 3B). In the case of CrkII, similar enhancement was observed by NGF, TGF-β, IL-2, and LPS (Supplementary information, Figure S7). In fact, the primary DNA sequence of the *Crk* promoter region contains a corresponding transcription binding sequence for AP-1, Smad, STAT5, and NF-κB (Supplementary information, Figure S8). Of several growth factors that enhance CrkI expression, TGF-β increased CrkI most effectively, and TGF-β plays a central role for EMT, thus we further investigated the relationship between TGF-β and Crk.

### Crk induced expression of TGF-β that augmented Crk-induced EMT

**Figure 4:**
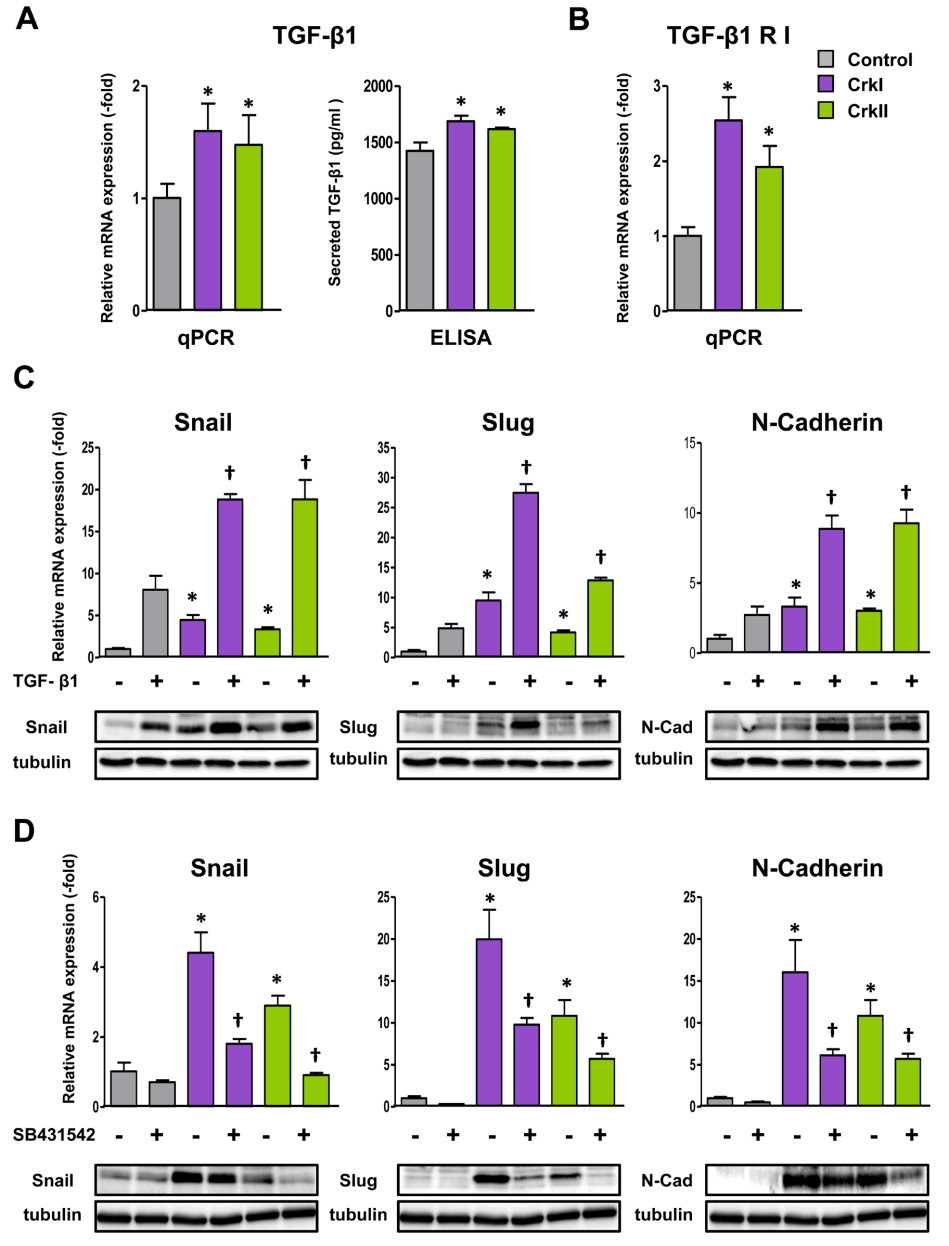
CrkI and CrkII upregulate the TGF-**β**1 signaling pathway through Rac1 and RhoA activation **A.** Increase of TGF-β1 expression as measured by qRT-PCR and ELISA in CrkI- and CrkII-expressing A549 cells. *P < 0.05 *versus* control cells. **B.** Increase of TGF-β receptor I (RI) expression in CrkI- and CrkII-expressing A549 cells. *P < 0.05 *versus* control cells. **C.** Effect of TGF-β1 on Crk-induced EMT. qPCR analysis of mRNA expression of Snail, Slug, and N-cadherin in CrkI- and CrkII-expressing A549 cells with 5 ng/mL of TGF-β1 treatment for 48 h. *P < 0.05 *versus* control cells. ^†^*P* < 0.05 *versus* treated control cells. Immunoblot analysis of indicated proteins are displayed at the bottom. Tubulin was used as an internal control. **D.** Effect of TGF-βRI inhibitor on Crk-induced EMT. qPCR analysis of mRNA expression of Snail, Slug, and N-cadherin in CrkI- and CrkII-expressing A549 cells with treatment of 10 μM TGF-βRI inhibitor (SB431542) for 48 h. *P < 0.05 *versus* control cells. ^†^*P* < 0.05 *versus* treated control cells. Immunoblot analysis of indicated proteins are displayed at the bottom. Tubulin was used as an internal control.

To examine whether an autocrine loop of TGF-β/Crk axis functions to facilitate EMT, we analyzed Crk-expressing A549 cells and confirmed the presence of elevated levels of TGF-β by qPCR and ELISA (Figure 4A) together with its receptor expression (Figure 4B). As a synergistic effect of TGF-β and Crk on EMT, TGF-β stimulation enhanced CrkI- and CrkII-dependent increases in the expression levels of Snail, Slug, and N-cadherin at both the mRNA and protein levels (Figure 4C). In addition, a decrease in E-cadherin and an increase in fibronectin together with a promotion of MMP2 activity were also observed following TGF-β stimulation (Supplementary information, Figure S9A-S9B).

Furthermore, TGF-β inhibitor, SB431542, suppressed CrkI- and CrkII-dependent elevation of those EMT-associated molecules at the mRNA (Figure 4D) and protein levels (Supplementary information, Figure S10). Inhibitors of Rac1 and ROCK were also confirmed to suppress expression of TGF-β and its receptor (Supplementary information, Figure S11). These results suggest the novel collaboration between TGF-β and Crk to induce EMT in human lung cancer cells.

### Overexpression of Crk at the invasive front of human lung cancer tissues

**Figure 5:**
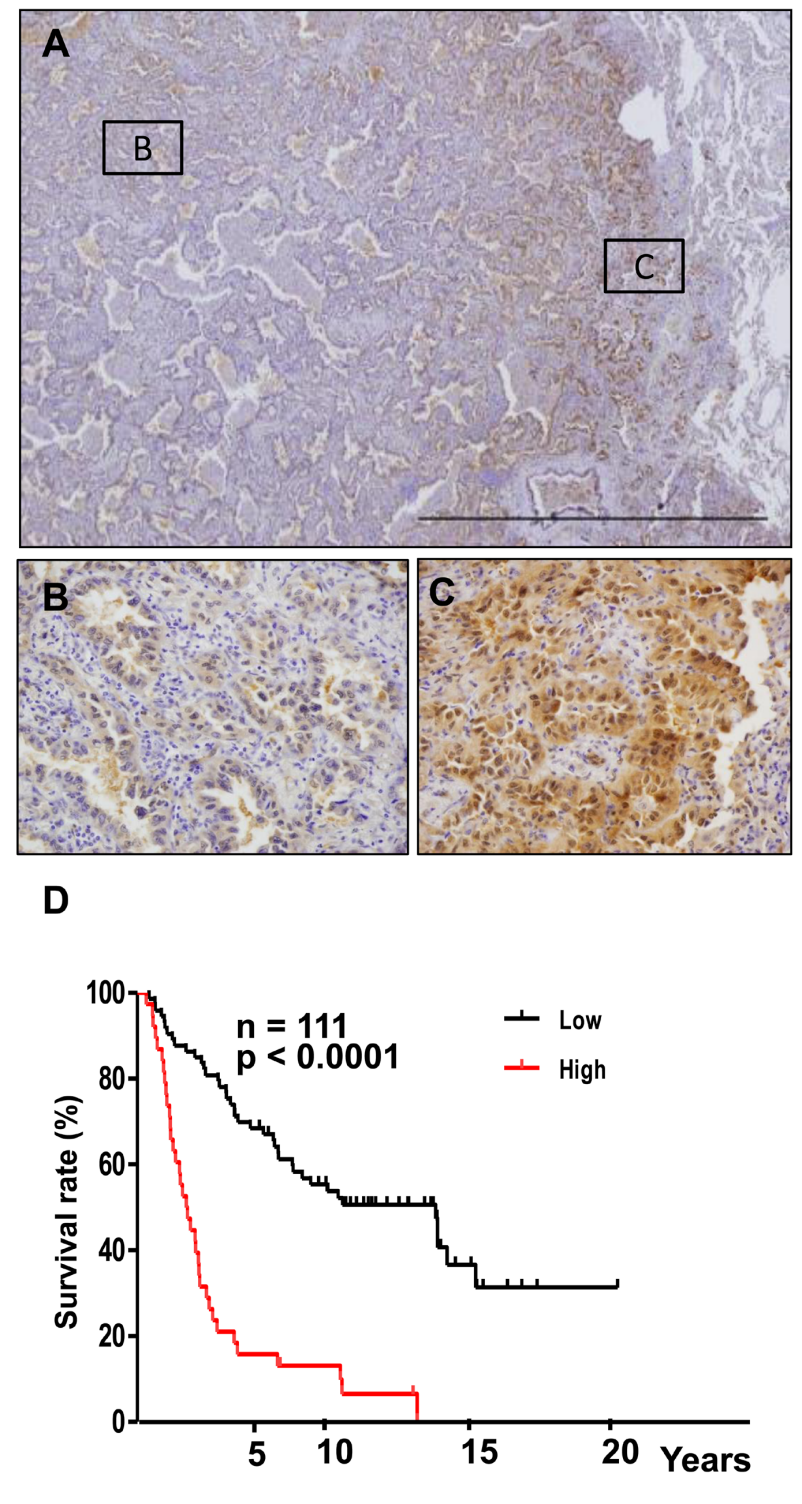
Overexpression of Crk at the invasion front of human lung cancer tissues **A.** Immunohistochemical analysis of Crk at the invasive front of lung cancer samples. Representative images of lower magnification. **B.** Magnified view at center area of the tumor outlined as square B in figure 5A. **C.** Magnified view at invasive area of the tumor outlined as square C in figure 5A. **D.** Kaplan-Meier analysis of overall survival of lung cancer patients. Cases with lower (black) and higher expression (red) of Crk are plotted.

To confirm the clinical significance of Crk-induced EMT, surgically resected human non-small cell lung cancer specimens were analyzed. We observed both cytoplasmic and nuclear staining in these specimens as previously reported [17, 21], however the staining was much more dominantly in the cytoplasm rather than the nucleus. Crk overexpression was observed at the invasive front of the tumor tissues by immunohistochemistry (IHC) (Figure 5A–5C). Furthermore, higher Crk expression was associated with a poor outcome in overall survival (Figure 5D). In IHC analysis, reciprocal decreases in E-cadherin could also be observed at the invasive front (Supplementary information, Figure S12A). In the center of the tumors, increased levels of E-cadherin were observed (Supplementary information, Figure S12B). Thus, evaluation of Crk at the invasive front of surgically resected tumor tissue may have prognostic value in non-small cell lung cancer patients in the future.

## DISCUSSION

To date, Crk signaling adaptor protein has been shown to be involved in various human cancers including lung cancer, ovarian cancer, bladder cancer, sarcoma, and brain tumors [15, 16, 18, 20]. In these human cancer tissues, Crk was basically overexpressed confirmed by IHC, and knockdown of Crk by shRNA in the corresponding cells was shown to suppress tumor malignancy. However, the regulatory mechanism of Crk has long been unknown. Recently, miR-126 has been reported to control *Crk* mRNA levels [22], but this may not be the universal mechanism because miR-126 has multiple target genes [23]. In fact, miR-126 did not significantly suppress Crk at the protein level in our hands (data not shown).

Tumor cell growth has been shown to be regulated by surrounding fibroblasts and inflammatory cells including macrophages comprising the tumor microenvironment, where cellular communications were dictated by growth factors and cytokines [11]. The primary genome sequence of the *Crk* promoter contains binding consensus sequences for several cytokine-dependent transcription factors, including 10 putative Smad binding elements (Supplementary information, Figure S8) [24–26]. In fact, *Crk* promoter activity was upregulated by several growth factors including TGF-β.

Considering tumor malignancy as invasion and metastasis, acquisition of mesenchymal features in carcinoma, namely EMT, is one of the central phenomena in which TGF-β may play a central role [10, 13, 27]. Here, we present autocrine activation of EMT through the TGF-β/Crk axis, and now hypothesize that in the initial stage of human cancer, each tumor cell has been exposed to various stimuli secreted from the fibroblasts or macrophages, and among them, TGF-β may enhance Crk levels in an individual cancer cell and lead to an EMT phenotype together with expression of both TGF-β and its receptor.

As the tumor microenvironment can regulate stemness and therapeutic resistance of cancer cells [14], we determined that cancer cells with Crk-induced EMT did not possess enhanced levels of stem cell markers including *Nanog*, *Sox2*, *Oct3/4*, and *CD44* (Supplementary information, Figure S13). It should be noted that the receptor tyrosine kinase MET was phosphorylated by CrkI (Supplementary information, Figure S4), consistent with a previous report that Crk may lead to morphological changes of MDCK epithelial cells through HGF/MET [28].

Involvement of small GTPases in TGF-β-dependent EMT has been reported. Rac1 has been shown to be mediated by TGF-β-induced MMP3 production [29]. Suppression of Rho is supposed to be involved in the early stage of TGF-β response to degrade tight junctions [30], and subsequent lamellipodia formation by activated Rho may function in cell motility, which positively contributes to EMT [31]. Indeed, phosphoproteomic analysis of TGF-β response showed time-specific activation of ROCK in skin cancer cells [32]. As Crk constitutively forms a complex with GEFs that activate small GTPases, we examined the role of Rac1 or RhoA in EMT using Rac1 and ROCK inhibitors. As TGF-β mediated Rac1/RhoA activation was reported to be involved in cancer metastasis [33], these inhibitors can be possible therapeutic reagents.

**Figure 6:**
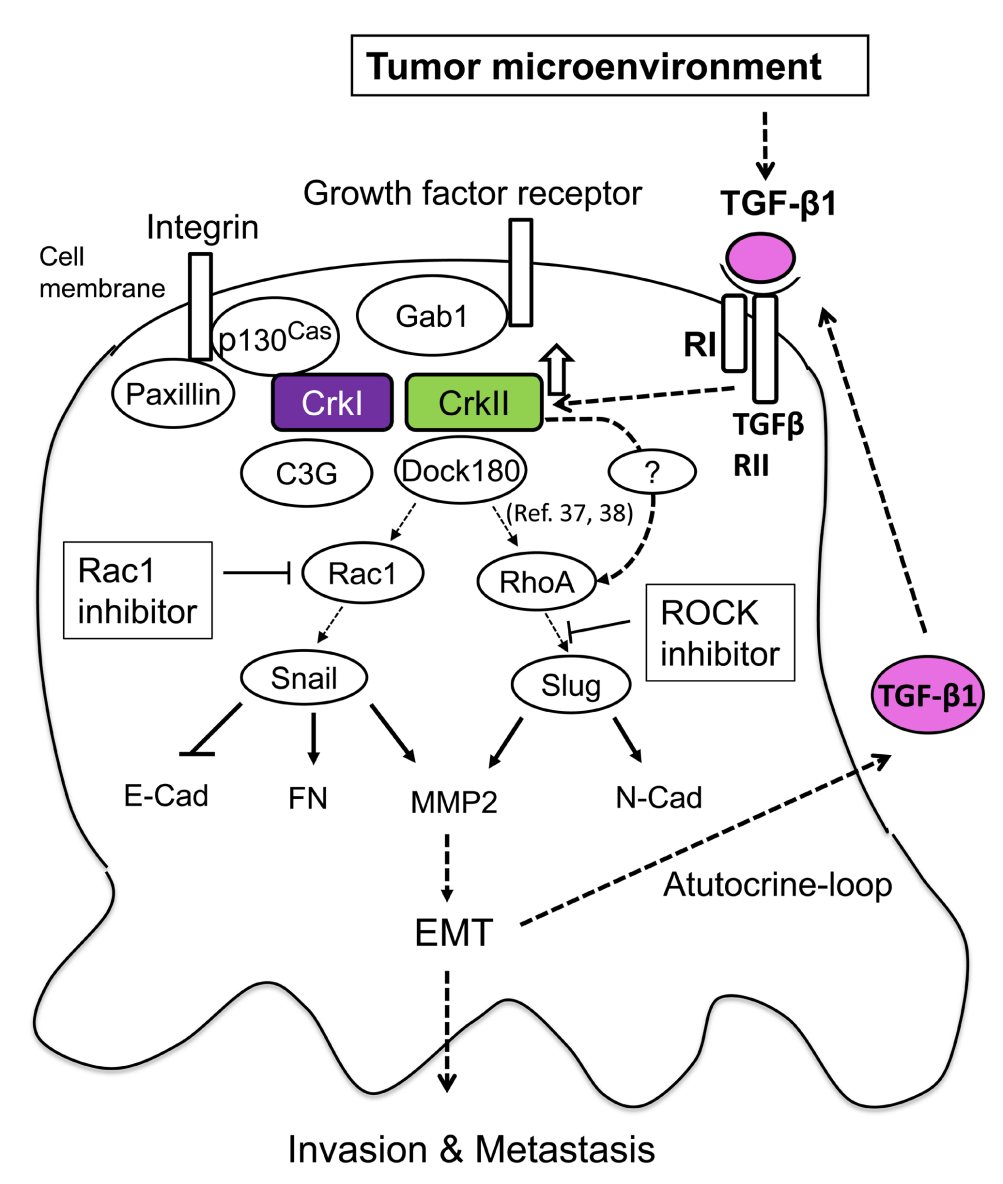
Schematic of the signaling mechanism of collaboration between TGF-β and Crk to induce EMT in human cancer cells TGF-β initially produced by cancer-associated fibroblasts may enhance expression of CrkI and CrkII, which leads to activation of Rac1 and RhoA to increase the levels of EMT regulators Snail and Slug. This leads to a cadherin switch demonstrating a decrease in E-cadherin and increase in N-cadherin expression, concurrent with MMP2 activation, resulting in the induction of EMT. Upregulation of CrkI- and CrkII-induced TGF-β production and its receptor in cancer cells forms a positive feedback loop for malignant progression.

To explore the mechanisms that Crk-induced Rac1 activation followed by snail expression, we examined i) the effect of NSC23766 on Dock180 induced Rac1 activation, ii) presence of Crk-mediated upregulation of Tiam/Trio, and iii) the effect of C21, which specifically inhibit Dock 5. Although NSC23766 has not been shown to affect Rac activation mediated Dock family members, we showed that NSC23766 blocked Rac1 activation partially in Dock180/Elmo overexpression cells (Supplementary information, Figure S14). Similar inhibitory effect of NSC23766 has been previously reported in Dock3 overexpression cells [34]. Because we could not demonstrate the significant increase of *Tiam* or *Trio* mRNAs both in CrkI and CrkII expressing cells, indirect pathway of Crk-Dock180-Rac-Trio/Tiam-Rac1 activation was not demonstrated in this study (data not shown). Furthermore, as C21 did not inhibit Rac1 activation in Crk overexpression cells, Crk-Dock5-Rac1 activation seems not to exist in these cells. Although the inverse activity relationship between Rac and RhoA remains to be established [35, 36], both Rac1 and RhoA activation seems to exist in Crk overexpressing lung cancer cells through Dock families [37, 38] or other molecules such as Src, Fak, and Abl those can be activated by Crk. Along with canonical TGF-β signaling through Smad-dependent transcriptional regulation, Crk-dependent EMT though Rac1 and RhoA activation may play a role in cancer progression (Figure 6).

Considering the EMT-induction potential of CrkI and CrkII, CrkI seemed to possess a higher ability in our study, consistent with the results of structural analysis of Crk, where CrkI simply comprises SH2-SH3 domains and constitutively binds to its up- and downstream targets to continuously generate active signals; in contrast, CrkII can be phosphorylated, with the subsequent conformational change leading to its self-inhibitory form [39]. As the mRNA of CrkI is generated by splicing of CrkII mRNA [40], and splicing regulators such as ESRP1 and SRSF1 have been reported to be involved in EMT [41–44], this underlying regulatory machinery is interesting and should be analyzed in the future.

Higher Crk expression at the invasive front of cancer tissues, as measured by immunohistochemistry of surgical specimens, may reflect the specific effect of the interaction between cancer cells and stromal tissues. In addition to a previous report describing an association between Crk expression and higher clinical stage [17], this study also demonstrated the poor prognosis of those who exhibit higher expression of Crk at the invasive front, thus, IHC evaluation of Crk may become a diagnostic tool to predict outcome of cancer patients.

This study delineates the novel spatiotemporal regulation of initiation and progression of cancer cells, where the initial step of the acquisition of EMT is most likely regulated by TGF-β produced by the tumor microenvironment, and in the process of subsequent cancer progression, a novel collaboration between TGF-β and Crk forms a positive feedback loop that may play a role especially at the invasive front of cancers. Thus, disruption of this TGF-β/Crk axis may become an effective target of cancer therapy in the future.

## MATERIALS AND METHODS

### Cells

A549 human lung adenocarcinoma cell line *was obtained from Riken* Cell Bank (Tsukuba, *Japan*) and cultured in *RPMI1640* (Nissui Pharmaceutical, Tokyo, Japan) supplemented with 10% fetal bovine serum (FBS), 1% penicillin, 1% streptomycin, and 1% L-glutamate. Human embryonic kidney 293T cells (Riken Cell Bank) were cultured in Dulbecco's modified Eagle's medium (Wako, Tokyo, Japan) supplemented with 10% FBS, 1% penicillin, 1% streptomycin, and 1% L-glutamate. For trans-endothelial invasion assays, HUVEC human umbilical vein endothelial cells (Riken Cell Bank) were cultured in endothelial cell basal medium (EBM-2) (Lonza, Walkersville, MD, USA) supplemented with FBS, hydrocortisone, hFGF-B2, VEGF, R3-IGF-1, ascorbic acid, hEGF, GA-1000, and heparin according to the manufacturer's instructions.

### Establishment of CrkI- and CrkII-overexpressing A549 cells

A549 cells were infected with lentiviruses for protein expression of CrkI and CrkII. First, 293T packaging cells were transfected either with pCX4-Flag-empty vector-puromycin (puro), pCX4-Flag-CrkI-puro, or pCX4-Flag-CrkII-puro. pCX4-GFP-bleomycin was co-transfected with these vectors using FuGENE HD (Promega, Madison, WI, USA). At 48 h after transfection, culture media containing lentiviral particles were collected and added directly to subconfluent A549 cells. Cells were selected by 0.5 μg/mL of puromycin (Calbiochem, La Jolla, CA, USA) and 1 mg/ml of Zeocin (Invitrogen, Carlsbad, CA, USA).

### Reagents

Recombinant human transforming growth factor β1 (TGF-β1) and SB431542 TGF-β1 receptor inhibitor were purchased from Sigma-Aldrich (St Louis, MO, *USA)* and diluted in distilled water or DMSO, respectively. NSC23766 Rac1-specific inhibitor and Y27632 ROCK-specific inhibitor were purchased from Calbiochem (San Diego, CA, USA).

### Quantitative real-time PCR

Total cellular RNA was isolated using the RNeasy Mini Kit (Qiagen, Valencia, CA, USA), and 1 μg of RNA was converted into cDNA by reverse transcription (RT) using Superscript VILO (Invitrogen, Carlsbad, CA, USA). The resulting cDNA was used for quantitative real-time PCR using SYBR Green DNA polymerase (Applied Biosystems, Warrington, UK). The sequences of primers are provided in Supplementary information, Table S1.

### Immunoblotting and immunoprecipitation

Immunoblotting and immunoprecipitation were performed by the standard procedure described elsewhere. Briefly, cells were lysed with lysis buffer containing 0.5% NP-40, 10 mM Tris-HCl (pH7.4), 150 mM NaCl, 1 mM EDTA, 50 mM NaF, 1 mM PMFS, 1 mM Na_3_VO_4_, and protease inhibitor mixture. The whole cell extracts were clarified by microcentrifugation at 15,000 rpm for 15 min at 4°C. Total protein lysates were subjected to immunoprecipitation or separated by SDS-PAGE for immunoblotting. Primary antibodies for immunoblotting are listed in Supplementary information, Table S2. Secondary antibodies labeled with peroxidase were used and the positive signal was developed using ECL detection reagent (GE Healthcare, Buckinghamshire, UK), followed by image analysis using an Imagequant LAS4000 mini (Fujifilm, Tokyo, Japan).

### Luciferase reporter assay

Cells were seeded in a 12-well plate at a density of 5 × 10^5^ cells/well and were transfected with the luciferase reporter plasmid (1 mg per well) and the thymidine kinase promoter-driven Renilla luciferase plasmid pRL-TK (20 ng per well) (Promega, Madison, WI, USA). After 24 h, cells were serum starved for 16 h, followed by stimulation with several growth factors and cytokines for 1 h. The cells were lysed and luciferase activity was measured with a Dual Luciferase Assay Kit (Promega). The activity of firefly luciferase was normalized by that of Renilla luciferase.

### ELISA for TGF-β1

The levels of secreted TGF-β1 in the conditioned medium were analyzed using the TGF-β1 immunoassay Quantikine ELISA (R&D Systems, Abingdon, UK) according to the manufacturer's instructions.

### Immunofluorescence

Cells were seeded at a density of 1 × 10^5^ in 35-mm glass bottom dishes (IWAKI, Newport, UK) and cultured overnight in RPMI medium. The cells were then fixed in 3% paraformaldehyde for 15 min, permeabilized with 0.1% Triton X-100 for 4 min, and blocked with 1% BSA for 20 min. Cells were incubated with anti-paxillin antibody (Ab) (BD Transduction Laboratories) overnight at 4°C, and then with a secondary antibody conjugated with AlexaFluor488 (Invitrogen) for 1 h at room temperature. F-actin was visualized by phalloidin conjugated with AlexaFluor594. GST-PAK-RBD was used as a probe to detect the active form of Rac, followed by incubation with an anti-GST antibody. Images were acquired using a confocal laser-scanning microscope (FV-300; Olympus, Tokyo, Japan).

### Trans-endothelial invasion assay

Human umbilical vein endothelial cells (HUVEC) (Riken Cell Bank) were seeded at a density of 5×10^4^ on 8-μm pore-size Matrigel-coated chambers (BD BioCoat), as per the manufacturer's instructions, in EBM-2 endothelial basal medium to form a monolayer on the upper chamber of the inserts, and incubated at 37°C in 5 % CO_2_ for 2 days. After confirmation of HUVEC cell monolayer formation by microscopy, the EBM-2 was carefully removed and 3×10^4^ stably GFP-expressing A549 control, A549 overexpressing CrkI, and A549 overexpressing CrkII cells were seeded into the upper chamber in serum-free RPMI medium. RPMI containing 10 % FBS to induce chemotaxis was added to the lower chamber. After incubation for 24 h at 37°C in 5 % CO_2_, the remaining cells were removed by scraping the upper chamber. The cells that had invaded through the HUVEC cell monolayer to the lower side of the inserts were counted by fluorescence microscopy (Keyence Biorevo BZ-9000, Keyence, Tokyo, Japan). The cells in full visual field (FF) (×200) per filter were counted.

### Mice tail vain injection experiments

All *in vivo* experiments were sanctioned by the Hokkaido University Ethics Committee for animal experiments. *1*×*10*^6^ cells of A549 stably expressing GFP with either empty vector as control or CrkI or CrkII were injected into the tail veins of eight BALB/cAJcl-nu/nu NOD mice per group; after 28 days, mice were sacrificed and total lung was preserved, whereupon fluorescent images of total lungs were immediately captured by fluorescence microscopy using a Keyence Biorevo BZ-9000 microscope (Keyence, Tokyo, Japan).

### Immunohistochemistry

This study was approved by the Medical Ethics Committee of Hokkaido University Hospital. In total, 111 patients (aged 64±9 years; sex, 36 were female and 75 were male) who received surgical resection for NSCLC were enrolled in this study. Surgically resected lung cancer samples were analyzed by IHC using an anti-Crk antibody that can recognize both CrkI and CrkII. The expression levels of these molecules were assessed according to proportion score (PS) and intensity score (IS) by two pathologists. PS was defined as follows: 0 (<1%), 1 (1–10%), 2 (11–80%), and 3 (81–100%), and IS was defined as follows: 1 (weakly positive), 2 (moderately positive), and 3 (strongly positive). Total score (TS) was represented by the sum of the PS and IS scores.

### Statistical analysis

The data represent the averages and standard deviations of experiments performed in triplicate. Statistical analyses were performed using the Student's *t*-test or one-way analysis of variance followed by the Newman–Keuls test as a post-test, using GraphPad Prism version 5.01 (GraphPad, San Diego, CA, USA). Survival analysis was carried out with Kaplan–Meier curves and the related log-rank tests. *P* values of <0.05 were considered statistically significant.

## SUPPLEMENTARY MATERIALS FIGURES AND TABLES


