# Supplementary material for: Novel signaling collaboration between TGF-β and adaptor protein Crk facilitates EMT in human lung cancer
Source: Oncotarget. 2016 Mar 24;7(19):27094–107. doi: 10.18632/oncotarget.8314 (PMC5053635; doi:10.18632/oncotarget.8314)
Supplement: Supplementary file 1 [file oncotarget-07-27094-s001.pdf]

Novel signaling collaboration between TGF-β and adaptor protein Crk facilitates EMT in human lung cancer

Supplementary Material

Supplementary Methods

Rac1 and RhoA Pull-down assay. Cells were grown to 70-90% confluence and then lysed with lysis buffer containing 1M HEPES (pH7.4), 5M NaCl, 0.5M EDTA, 10% NP40, 2M $MgCl_2$, 50% glycerol, PMSF and protease inhibitor mixture for Rac1, whereas 50 mM Tris-HCl (pH7.4), 150 mM NaCl, 1 mM EDTA, 1% NP-40, 30 mM $MgCl_2$, 10% glycerol, 1 mM DTT, PMSF, and protease inhibitor mixture for RhoA. 1 mg total protein lysate was incubated with 10 µg GST-PAK2 RBD for Rac1 and GST-rhotekin for RhoA with rotating for 30 min at 4°C, and then incubated with 20 µl glutathione sepherose 4B (Amersha Bioscience, Piscataway, NJ, USA) with rotating for 30 min at 4°C. After that supernatant was discarded and the pellet formed was washed with lysis buffer and recollected again and washed two times, final pellet was suspended in 20 µl of 2X SDS sample buffer and subjected into 12% SDS-PAGE gel, and immunoblotted for monoclonal mouse anti-Rac1 (BD Transduction Laboratories) and anti-RhoA (Santa Cruz) antibodies.

Wound healing assay. Cells were seeded at a density of 3×10^5 in 60 mm dishes and incubated in RPMI medium to grow to confluence. After that dishes were scratched using a pipette tip. The cells were washed twice with warmed PBS and incubated in RPMI medium and photographed every 6 hours. Cell migration was calculated by measuring the distance covered by migrating cells and further dividing by that of the original wound (wound closure %).

Transwell migration assay. The uncoated Boyden chamber for transwell assay (8 µm pore size, Corning Costar, Corning, NY, USA) was able to quantify chemotactic migration. 3×10^4 cells suspended in RPMI serum free medium were seeded into the upper chamber, RPMI medium with 10% FBS to induce chemotaxis was added into the lower chamber, and after 16 h of incubation at 37 °C, 5 % CO_2, the remaining cells were removed from the upper chamber by scrapping. The cells that had passed through to the lower side of the inserts were fixed with 100% methanol and stained

with 0.04% crystal violet. The total number of migrating cells was quantified by counting the amount of cells per 5 random 400x fields.

Matrigel invasion assay. Following the manufacturer's protocol, the 8 μm pore size, Matrigel coated chambers (BD BioCoat, Bedford, MA, USA) were allowed to defrost at room temperature and rehydrated with RPMI medium. 3×10^4 cells were then seeded into the upper chamber and incubated in serum free RPMI medium, 10% FBS supplemented RPMI medium was added into the lower chamber, after 24 h of incubation h at 37 °C, 5 % $CO_{2,}$ the remaining cells in the upper chamber were removed by scraping. The cells which had invaded through the matrigel coat to the lower side of the inserts were fixed with 100% methanol and stained with 0.04% crystal violet. The total number of invading cells was quantified by counting in 5 random fields (400× high power field).

Proliferation assays. Cellular proliferation assessment was done by bromodeoxyuridine (BrdU) cell proliferation ELISA assay (Roche, Mannheim, Germany) and MTT cell proliferation kit (Roche, Mannheim, Germany) as per manufacturer's guidelines. For growth curve, cells were seeded at a density of 3×10^5 in 60 mm dishes and incubated in RPMI medium with 10% FBS. The cells were counted in triplicate after 24 h, 48 h and 36h.

Gelatin zymography. Cells were cultured as previously described for RNA extraction, and conditioned medium was clarified by centrifugation at 1,500 rpm for 15 min. 20 μl of conditioned medium was mixed with an equal volume of 2× SDS sample buffer and subjected to SDS-PAGE containing 25 mg/ml gelatin. The gels were washed twice in 2.5% Triton X-100 in PBS for 15 min at room temperature, rinsed twice in with distilled water, and incubated at 37°C for 18 h in an incubation buffer containing 50 mM Tris, 5 mM $CaCl_2.H_20$ and 1 mM $ZnCl_2$. The resulting gel was stained and fixed with 50% methanol and 10% acetic acid containing 1.25 mg/ml Coomassie Brilliant Blue for 30 min and then destained with 10% methanol and 5% acetic acid. The zymogram was imaged using D70s Canon digital camera (Canon, Tokyo, Japan).

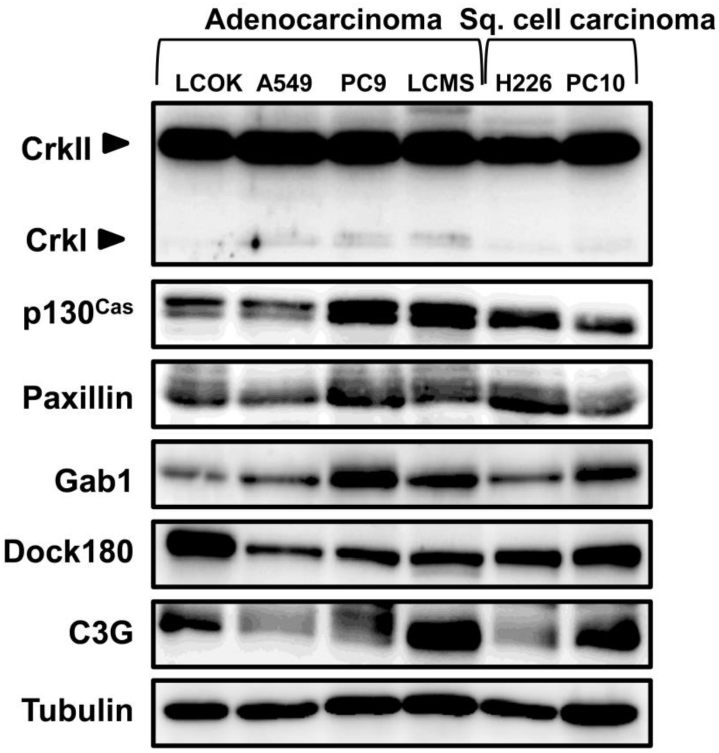

Supplementary Figure S1: Expression of Crk and its related proteins in various human lung cancer cell lines. Immunoblotting showed the levels of p130Cas, paxillin, Gab1, Dock180, and C3G in various human lung cancer cell lines, LCOK, A549, PC9, LC-MS as adenocarcinoma, and H226 and PC10 as squamous cell carcinoma. Tubulin was used as internal control.

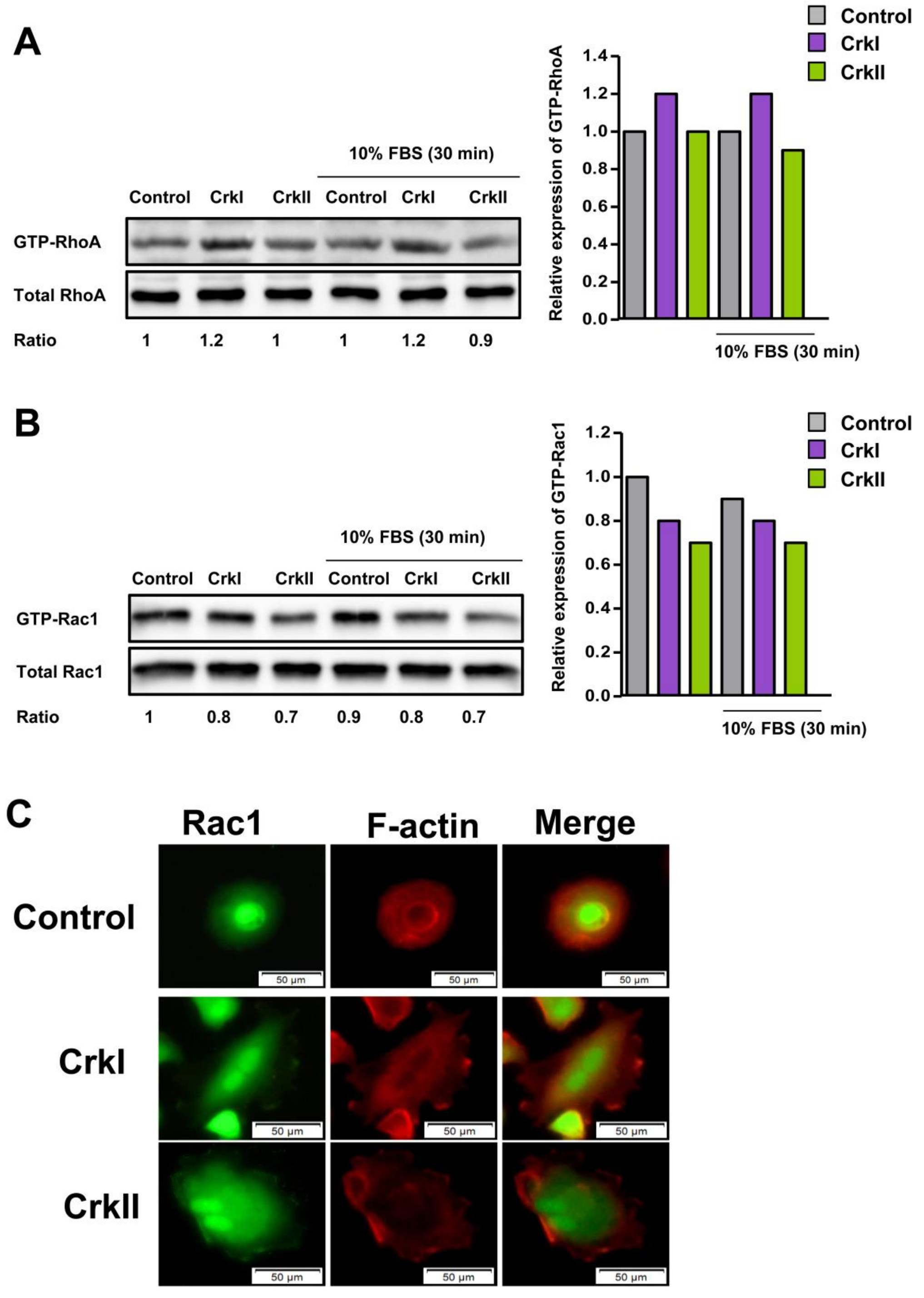

Supplementary Figure S2 : Activity of small GTPases RhoA and Rac1 in Crk knockdown A549 cells. (A)(B) Analysis of activation of Rho (upper panel) and Rac1 (lower panel) in Crk-expressing cells with serum starvation (left three lanes) and 1hr after 10% FBS stimulation (right three lanes) by pull down assay by using GST-Rhotekin-RBD and GST-PAK-RBD as probe. The amounts of GTP-Rho/Rac were normalized by total levels of Rho/Rac and compared as control as 1.0 and described at the bottom of each panel, and also displayed as bar graphs at right side. (C) Immunofluorescent analysis of active form of Rac1 probed with GST-PAK-RBD (green). Membrane ruffling was visualized by phalloidine for actin staining (red). Cells with Rac1-positive ruffling were counted as positive cells.

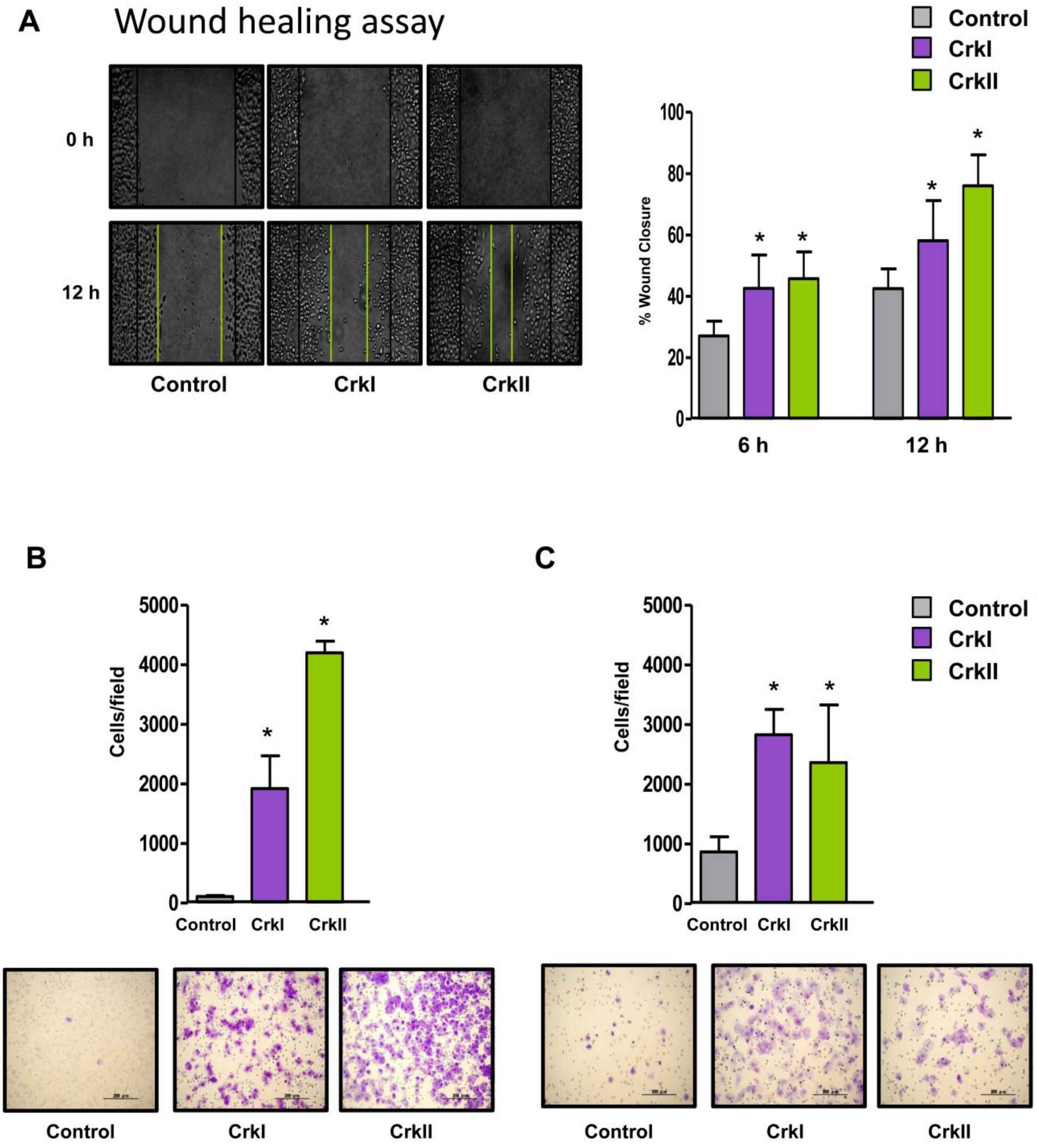

Supplementary Figure S3: Cellular motility and invasion potential of Crk knockdown A549 cells. (A)Wound-healing assay was performed and percentages of the closure at 6h and 12 h were indicated as bar graph (right). Representative photographs of wound closure at 12 h were presented (left panel). (B)Transwell migration assay. Representative photographs show the number of migrated cells through the uncoated filters after 16 h (bottom panels) and measured cell numbers were indicated as bar graph. (C)Matrigel-invasion assay. Representative photographs show the number of invaded cells through the matrigel-coated filters after 24 h (bottom panels) and measured cell numbers were indicated as bar graph.

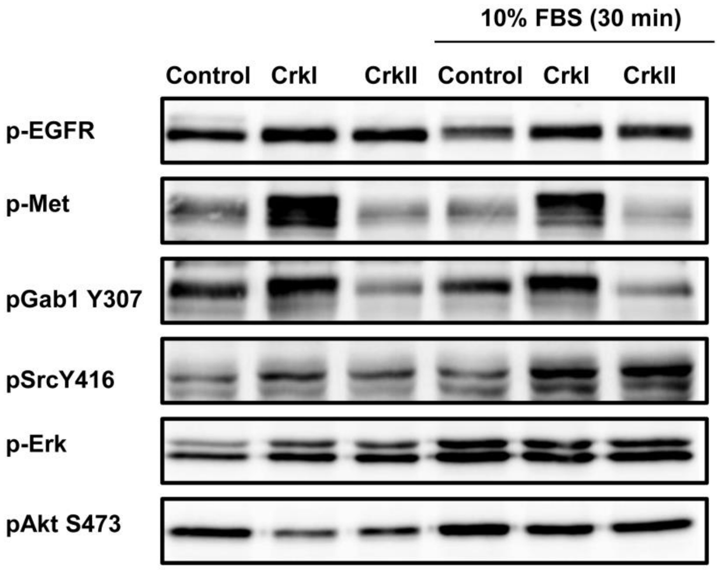

Supplementary Figure S4: Phosphorylation levels of tyrosine kinases, EGF, MET, Src and its related proteins, Gab1, Erk, and Akt. Immunoblotting of phosphorylation levels of receptor tyrosine kinases as EGFR and Met and their related proteins Gab, Src, ERK, and Akt in Crk-expressing A549 cells with serum starvation (left three lanes) and 1hr after 10% FBS stimulation (right three lanes).

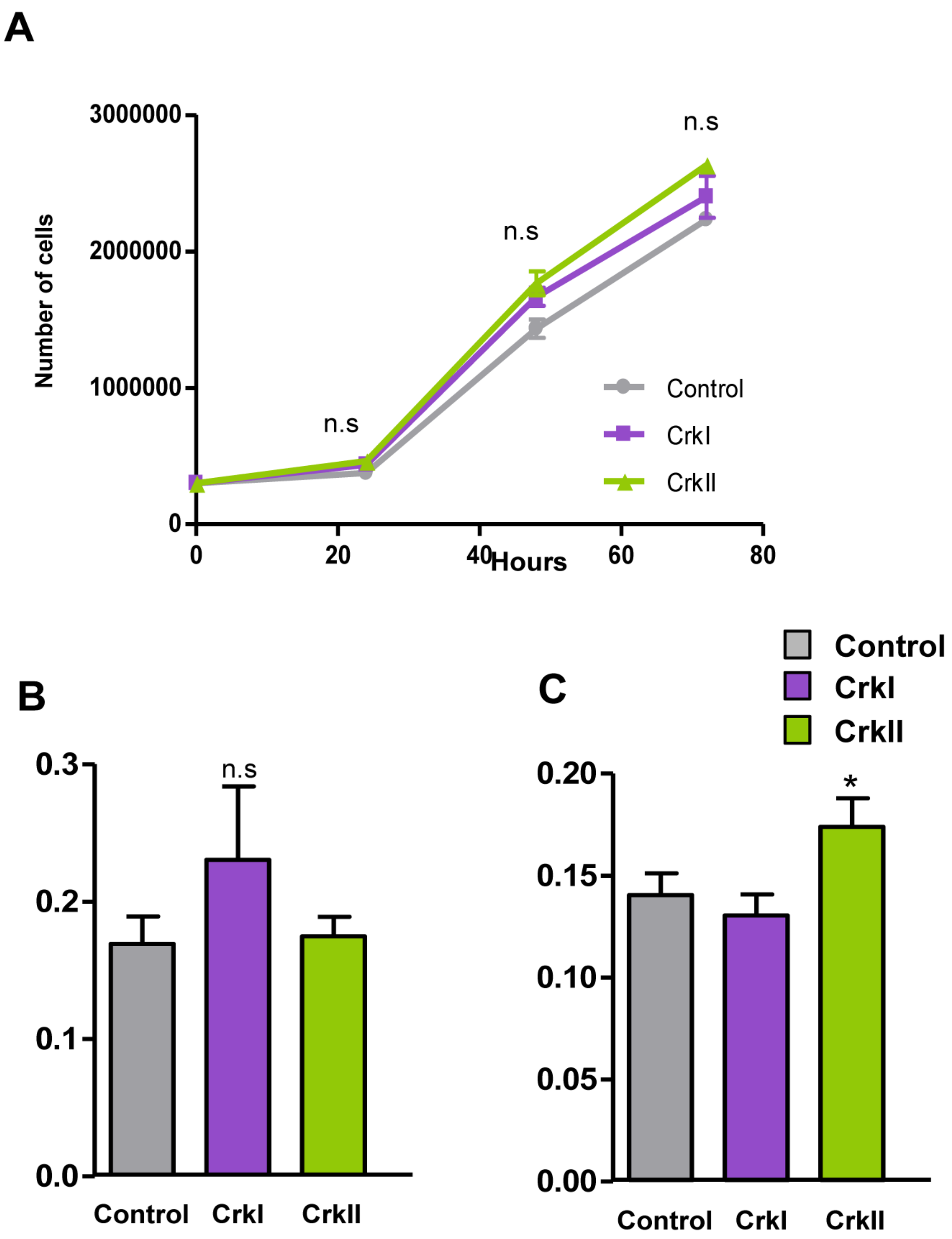

Supplementary Figure S5: Growth potential of Crk knockdown A549 cells. (A) Growth rates of A549 cells expressing CrkI or CrkII compared to control cells for 72 h. (B) BrdU assay represents the proliferation activity CrkI- and CrkII- expressing A549 cells. (C)MTT assay representing the proliferation activity of the CrkI- or CrkII-expressing A549 cells. N.S.: not significant. *p < 0.05. Values are means ± SD from 3 independent experiments

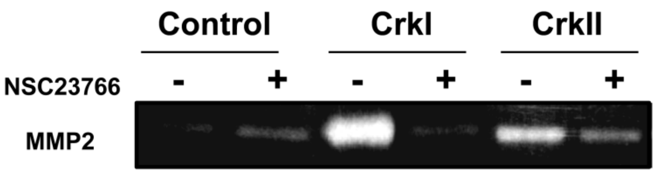

Supplementary Figure S6: Mesencymal features of Crk-expressing A549 cells. Zymography of gelatinase activity by MMP2 in Crk-expressing A549 cells and effects of Rac inhibitor (NSC23766).

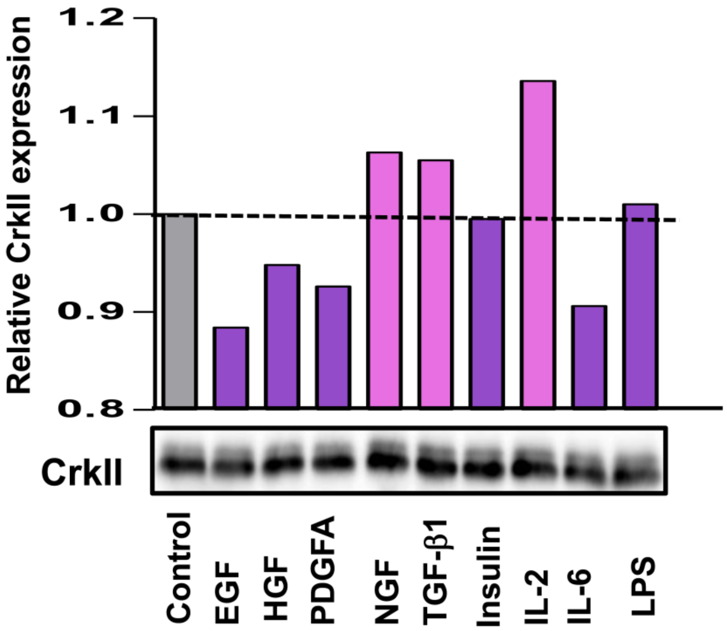

Supplementary Figure S7: Various stimuli enhanced CrkII expression. Serum starved A549 cells were stimulated with indicated growth factors and cytokines, and after 48 hrs, expression levels of CrkII were examined. The results of immunoblotting are displayed and the quantification of each lane is indicated as bar graph.

Crk promoter region

TGGAGTGTCAACAACAATGTCCTAGGTGAATCAATGAAAATGAGAACGTCA
GCTGGTCTGTGGAGAAGCTAGAGGAACGAGCAGAAACGGACCGACGTGG
CCGGGCGCGATGTTTCTCGCCTGTAATCCCAGCACTTTGGGAGGCCGAGGC
GGGCGGATCACCTGAGGTCAGGAGATTGAGACCATCCTGGCCGACATGGTG
AAACCCCCGTCTCTACTAAAAATACAAAAAACAGCCGGGCGCGGTGGCTCA
CGCCTGTAGTCTCAGCTACTCGGGAGGCTGACGCAGGAGAATCGCTTGAAC
CCGGGAGCAGTGAGCCGAGATCGCGCCATTGCACTCCAGCGTGGGCGACA
GAACAAGACTCCGTCTCAAAAAGAAAAAGAAAAAAGAAACAGACTGATATC
GCAGGAAGCCTGGCCGAGAACTCAGGCCCCAGGGCCACACGCATCTTTTCT
CCTCCAATTCTGTCTGGGAGTCGCTGCCTAGAAGTCCCAGGTCGTCCAGGAA
CCGGGGCCCAGACGAGAAGCACCGCTCTGTCCGCAGCGCAGGCGCGCGTC
TCTGCGGGAGGCTGCGAGGCCCCGCCCCTCGGGCCCTAGACCCCGCCTCCC
ATCCCCCGCAGCCCGGGCCCGGGAAGGGCGGCACCGCCCATGAATCCGCAT
GCGCAGTGCGCGCGCGAGGGCGCCTTGCCCTTCTCCCAATGGCGGGCCGC
GGGCCCCTTCCCGGCAGCCTCCCTCGGGCAGGGAGCGCGTCATTTCCGGAG
GGGGAGGCCCGCGGCTGCCGCCGCCATTTCGGGCGCTGCTGTGAAGCTGA
AACCGGAGCCGGTCCGCTGGGCGGCGGGCGCCGGGGGCCGGAGGGGCG
CGCGCGGCGGCGGCACCCCAGCGTTTAGGCGCGGAGGCAGCCATGGCGG
GCAACTTCGACTCGGAGGAG

Smad binding element (SBE).：AGAC, GGCGCC, GTCT

Supplementary Figure S8: Putative Smad biding element (SBE) in Crk promoter region. SBEs are indicated as red color. GC-rich SBE is indicated with under bar.

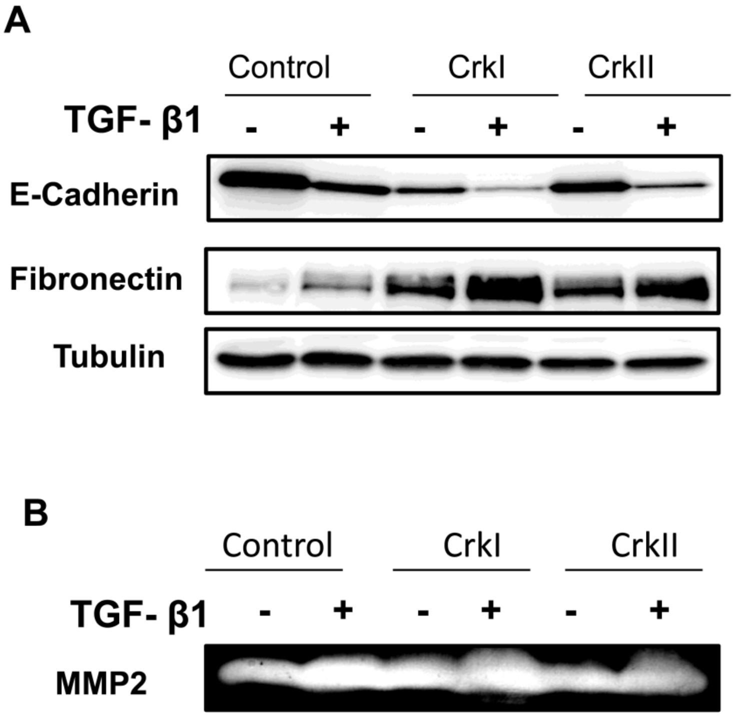

Supplementary Figure S9: Crk knockdown suppressed TGF-β induced mesenchymal features in A549 cells. (A) Enhancement of TGF-β for decrease of E-Cadherin and increase of fibronectin in A549 cells by immunoblotting. Tubulin is used as loading control. (B)Enhancement of gelatin zymography of MMP2 activity by TGF-β.

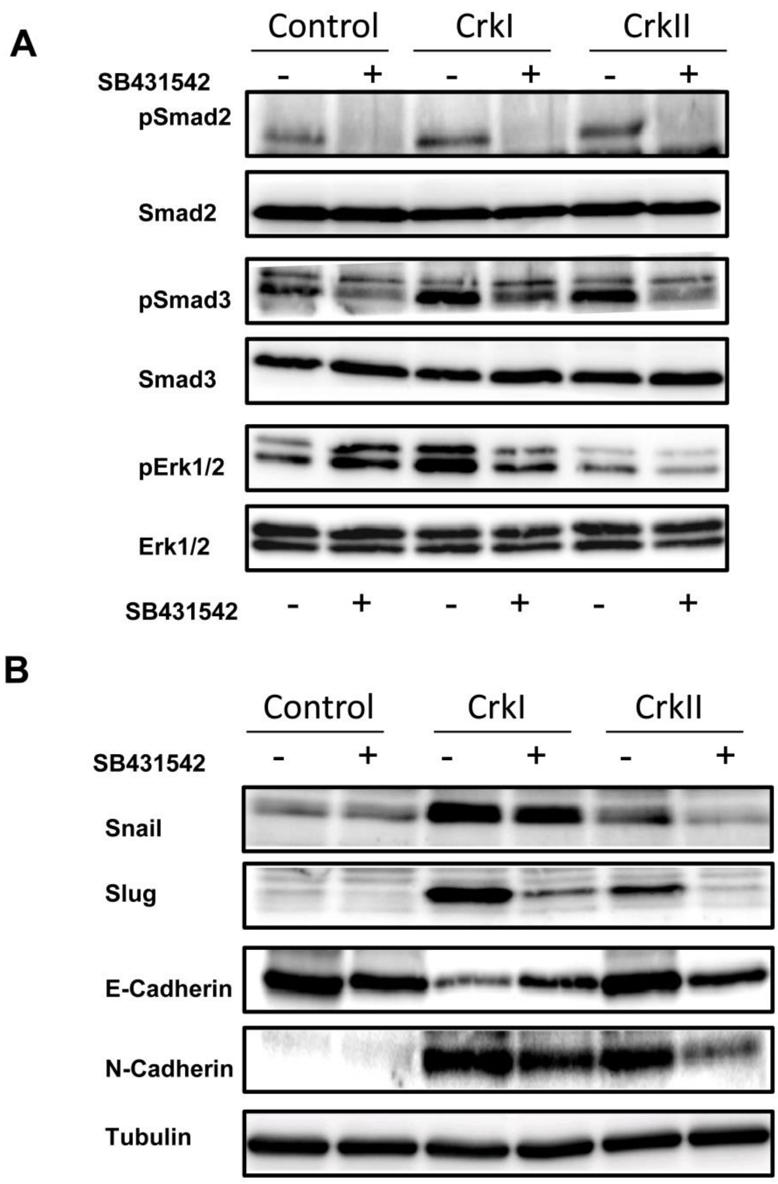

Supplementary Figure S10: A. Up-regulation of pSmad2 and pSmad3 by CrkI and CrkII and their inhibition by TGF-β receptor inhibitor. B. Increase of expression of Snail, Slug, N-Cadherin and decrease of E-Cadherin by CrkI and their inhibition by TGF-β receptor inhibitor.

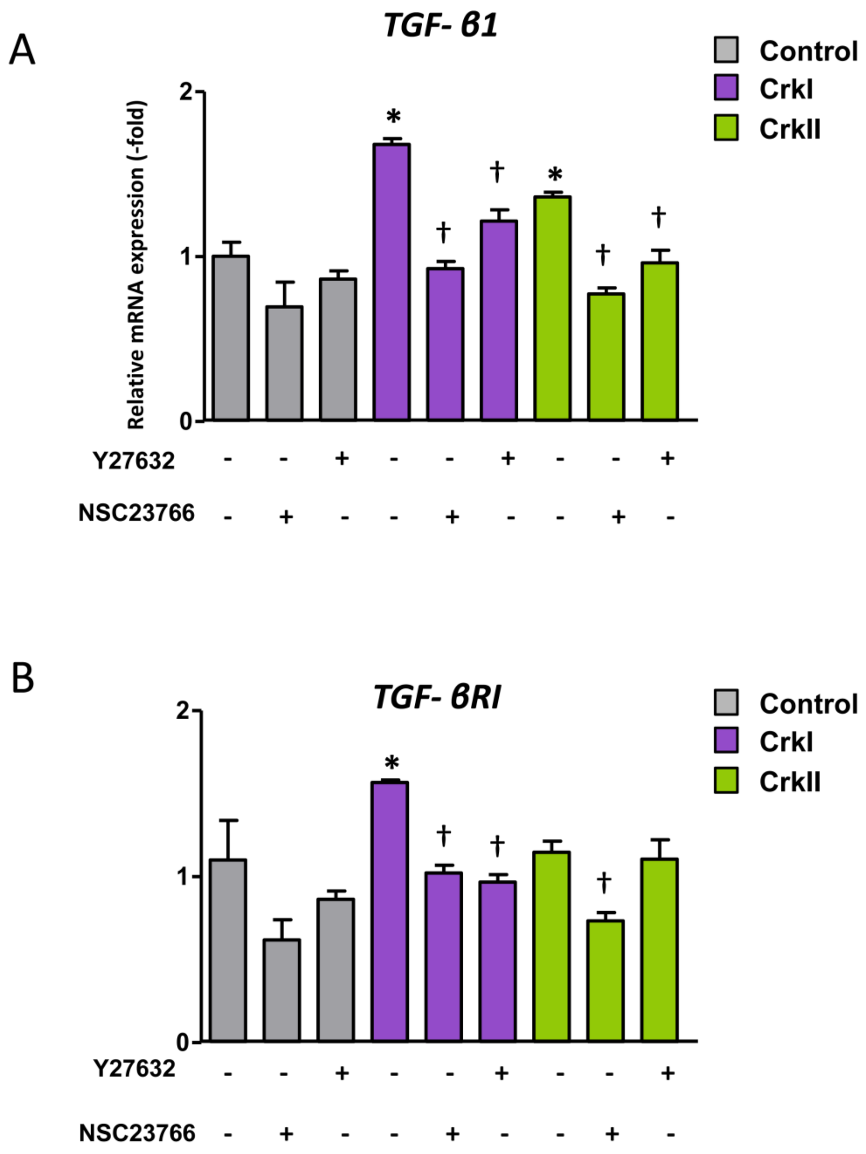

Supplementary Figure S11: Effects of Rac1 and ROCK inhibitors on TGF-β and its receptor.qRT-PCR analysis of mRNA expression of and TGF-β (A) and TGF-β receptor (B). Effects of ROCK inhibitor Y27632 (10 µM) and Rac1 inhibitor NSC23766 (50 µM) were also analyzed.

A

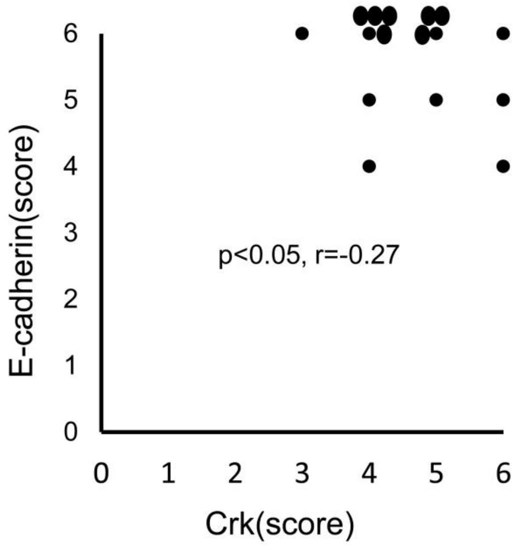

B

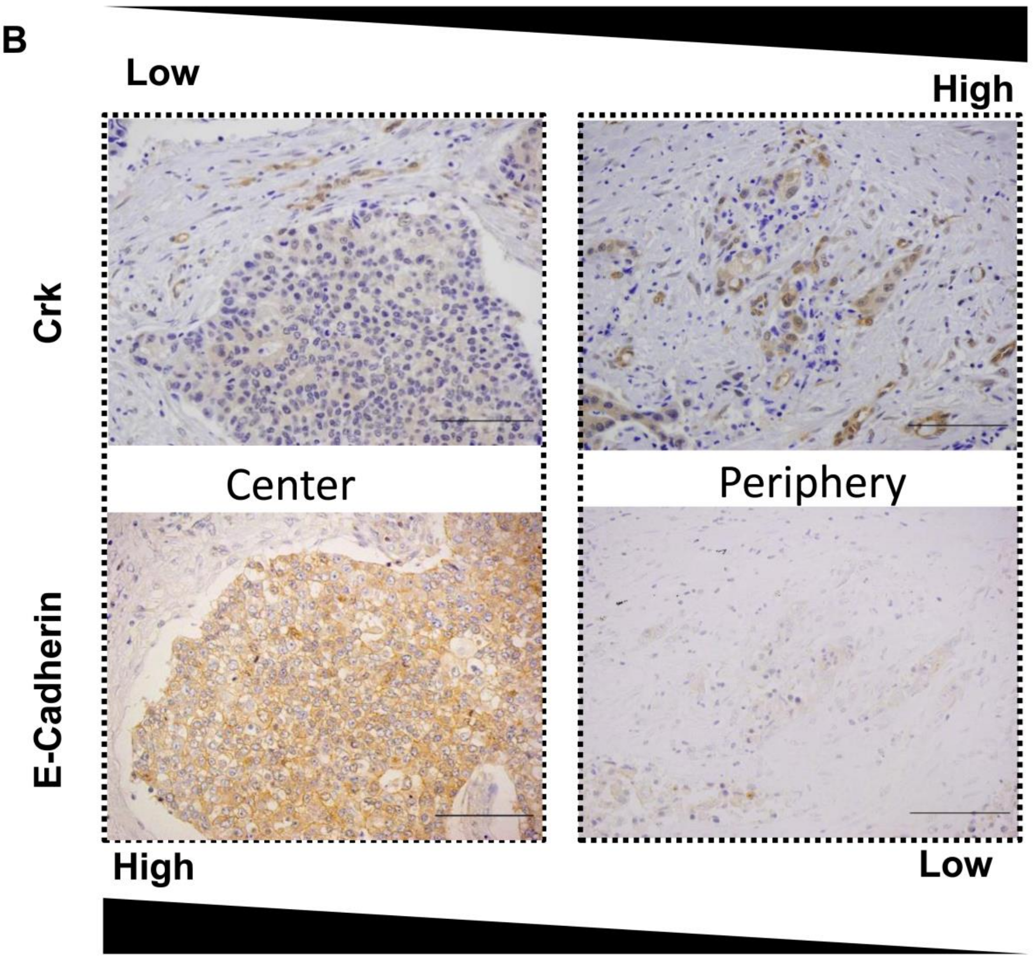

Supplementary Figure S12: Reciprocal expression of E-cadherin and Crk in human kung cancer specimens evaluated by immunohistochemistry. N=15 cases. (A) Representative photograph of expression of Crk and E-cadherin by IHC in center part and periphery of the identical tumor nodule. Bars indicate 50μm (B).

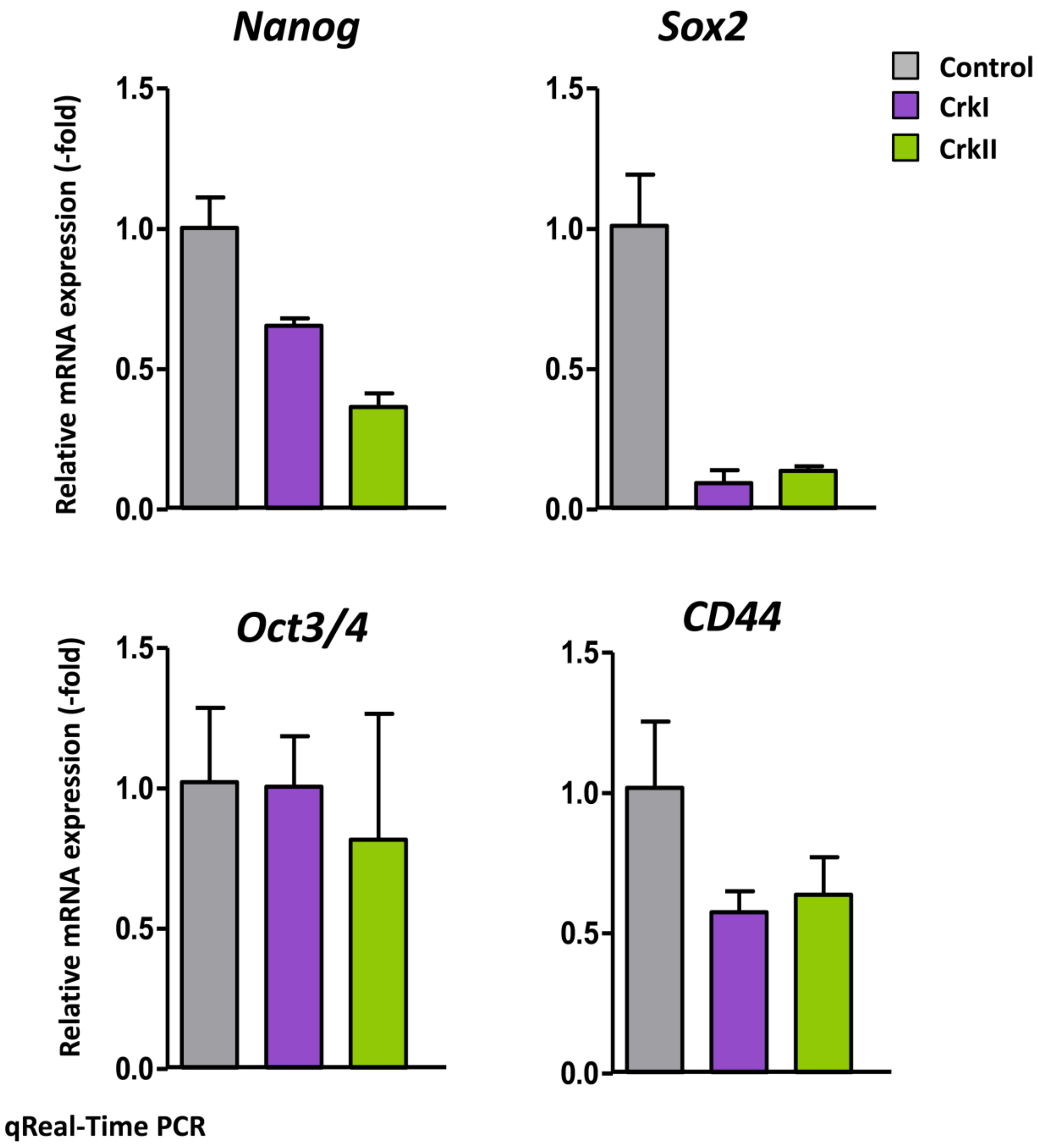

Supplementary Figure S13: qPCR analysis of stemness-related genes as Sox2, Nanog, Oct3/4, and CD44 in Crk-overexpressing A549 cells. Levels of control cells were arbitarily designated as 1.0 and displayed as graph with control cells (gray bar), and CrkI- (purple bar) and CrkII- (green bar) expressing cells.

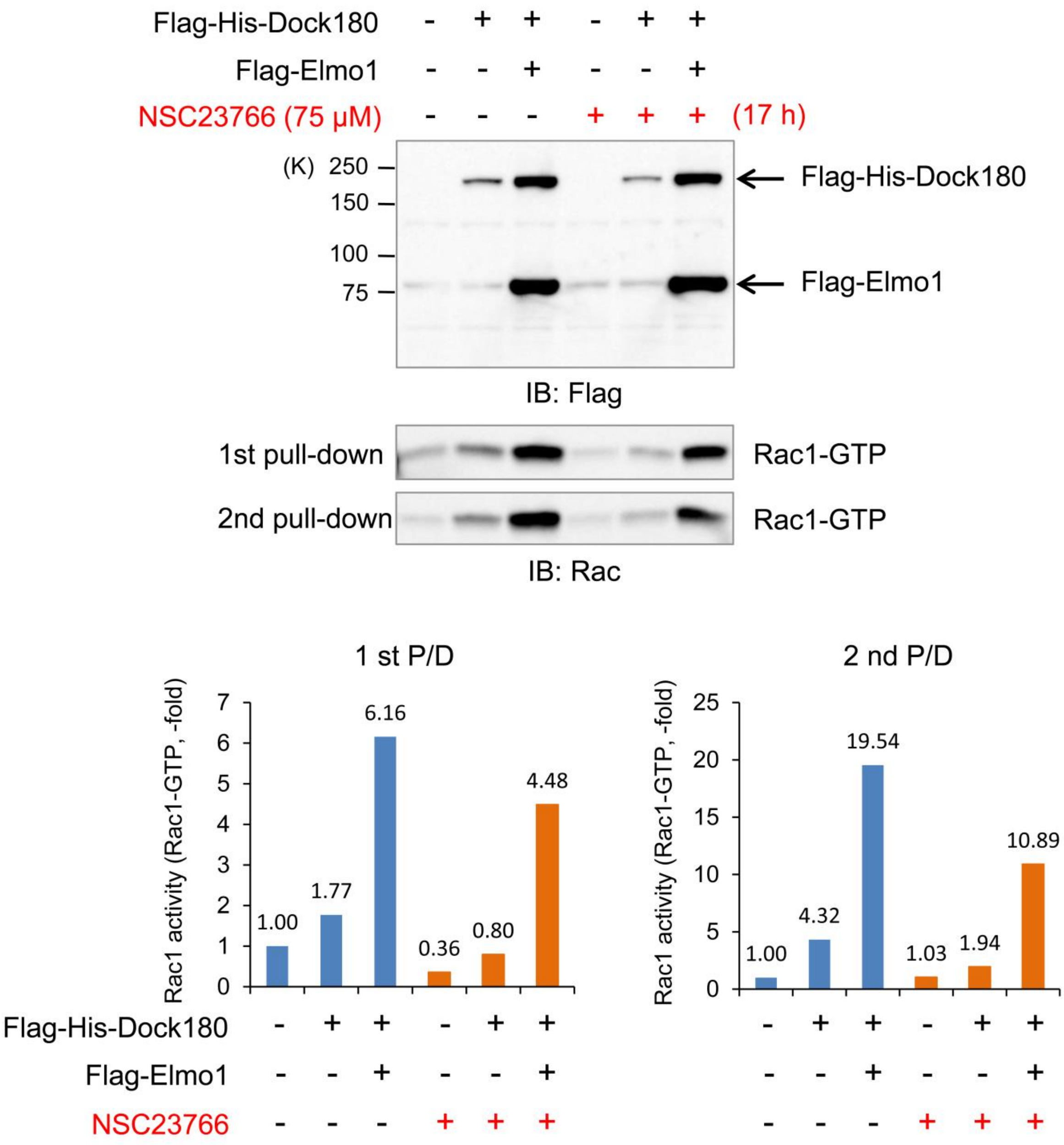

Supplementary Figure S14: Effect of Rac1 inhibitor NSC23766 (75 µM) on Dock180/ELMO induced Rac1 activation in 293T cells in independent two experiments.

Supplementary Table S1 Primers for qRT-PCR for EMT-associated molecules

GAPDH	F	5’-AGCCACATCGCTCAGACAC-3’
	R	5’-GCCCAATACGACCAAATCC-3’
TGF-β1	F	5’-GACTACTACGCCAAGGAGGTCA-3’
	R	5’-TGCTGTGTGTACTCTGCTTGAAC-3’
TGF-βRI	F	5’-GTGGCGGGGAGAAGAAGTTGCTGTTAAG-3’
	R	5’-CTCGCCGTGGACAGAGCAAGTTTTATC-3’
Snail	F	5’-GCTGCAGGACTCAATCCAGA-3’
	R	5’-ATCTCCGGAGGTGGGATG-3’
Slug	F	5’-TGGTTGCTTCAAGGACACAT-3’
	R	5’-GTTGCAGTGAGGGCAAGAA-3’
E-Cadherin	F	5’-TCCATTTCTTGGTCTACGCC-3’
	R	5’-CACCTTCAGCCATCCTGTTT-3’
N-Cadherin	F	5’-GTGCCATTAGCCAAGGGAATTCAGA-3’
	R	5’-GCGTTCCTGTTCCACTCATAGGAGG-3’
Fibronectin	F	5’-GTGTTGGGAATGGTCGTGGGGAATG-3’
	R	5’-CCAATGCCACGGCCATAGCAGTAGC-3’
MMP-2	F	5’-ATAACCTGGATGCCGTCGT-3’
	R	5’-AGGCACCCTTGAAGAAGTAGC-3’

F: Forward primer, R: Reverse primer

Supplementary Table S2 List of antibodies used in this study

Ab	species	company
Crk	mouse	BD Transduction Laboratories
Dock180	rabbit	Cell Signaling Technology
C3G	rabbit	Santa Cruz Biotechnology
p130^{Cas}	mouse	BD Transduction Laboratories
Gab1	rabbit	Upstate Biotechnology
Paxillin	mouse	BD Transduction Laboratories
phospho-Smad 2	rabbit	Cell Signaling Technology
Smad 2	rabbit	Cell Signaling Technology
phospho-Smad 3	rabbit	Cell Signaling Technology
Smad 3	rabbit	Cell Signaling Technology
phospho- ERK1/2	rabbit	Cell Signaling Technology
ERK1/2	rabbit	Cell Signaling Technology
phospho-AKT	rabbit	Cell Signaling Technology
AKT	rabbit	Cell Signaling Technology
phospho-GSK-3β	rabbit	Cell Signaling Technology
Snail	rabbit	Cell Signaling Technology
Slug	rabbit	Cell Signaling Technology
N-Cadherin	rabbit	Cell Signaling Technology
Fibronectin	mouse	BD Transduction Laboratories
E-Cadherin	rabbit	Cell Signaling Technology
Tubulin	mouse	Sigma Aldrich
phosphothyrosin-PY20	mouse	BD Transduction Laboratories
